# Synthesis and Characterization of Na-Zeolites from Textile Waste Ash and Its Application for Removal of Lead (Pb) from Wastewater

**DOI:** 10.3390/ijerph18073373

**Published:** 2021-03-24

**Authors:** Tabassum Hussain, Abdullah Ijaz Hussain, Shahzad Ali Shahid Chatha, Adnan Ali, Muhammad Rizwan, Shafaqat Ali, Parvaiz Ahamd, Leonard Wijaya, Mohammed Nasser Alyemeni

**Affiliations:** 1 Department of Applied Chemistry, Government College University Faisalabad, Faisalabad 38000, Pakistan; tabassumehussain@gmail.com; 2 Department of Chemistry, Government College University Faisalabad, Faisalabad 38000, Pakistan; abdullahijaz@gcuf.edu.pk (A.I.H.); chatha222@gmail.com (S.A.S.C.); 3 Department of Physics, Government College University Faisalabad, Faisalabad 38000, Pakistan; adnnan_1982@yahoo.com; 4 Department of Environmental Sciences and Engineering, Government College University Faisalabad, Faisalabad 38000, Pakistan; mrazi1532@yahoo.com; 5 Department of Biological Sciences and Technology, China Medical University, Taichung 40402, Taiwan; 6 Department of Botany and Microbiology, College of Science, King Saud University, Riyadh 11451, Saudi Arabia; pahmad@ksu.edu.sa (P.A.); lwijaya@ksu.edu.sa (L.W.); mnyemeni@ksu.edu.sa (M.N.A.)

**Keywords:** fly ash, Na-zeolite synthesis, adsorbent materials, Pb (II) removal, water treatment

## Abstract

Massive production of carcinogenic fly ash waste poses severe threats to water bodies due to its disposal into drains and landfills. Fly ash can be a source of raw materials for the synthesis of adsorbents. Rag fly ash as a new class of raw materials could be a cheap source of Al and Si for the synthesis of Na-zeolites. In this work, NaOH activation, via a prefusion- and postfusion-based hydrothermal strategy, was practiced for the modification of rag fly ash into Na-zeolite. Morphology, surface porosity, chemical composition, functionality, mineral phases, and crystallinity, in conjunction with ion exchangeability of the tailored materials, were evaluated by SEM, ICP-OES, XRF, FTIR, XRD, and cation exchange capacity (CEC) techniques. Rag fly ash and the synthesized Na-zeolites were applied for the removal of Pb (II) from synthetic wastewater by varying the reaction conditions, such as initial metal ion concentration, mass of adsorbent, sorption time, and pH of the reaction medium. It was observed that Na-zeolite materials (1 g/100 mL) effectively removed up to 90–98% of Pb (II) ions from 100 mg/L synthetic solution within 30 min at pH ≈ 8. Freundlich adsorption isotherm favors the multilayer heterogeneous adsorption mechanism for the removal of Pb (II). It is reasonable to conclude that recycling of textile rag fly ash waste into value-added Na-zeolites for the treatment of industrial wastewater could be an emergent move toward achieving sustainable and green remediation.

## 1. Introduction

The demand for textile goods is increasing exponentially around the globe due to population growth and modern living standards. Regrettably, the textile sector is creating many environmental challenges due to a lack of resources and improper waste management [1]. Hazardous fly ash discharged by textile industries and their discarding attitude poses the biggest foremost challenges, and it is a severe environmental issue around the globe [2]. Nowadays, researchers are utilizing fly ash waste for the synthesis of value-added adsorbent materials. The novel approach for utilizing fly ash waste into unique Na-zeolites contributes equally to modern solid waste management and the economical treatment of wastewater [3]. In several underdeveloped countries, there are deleterious consequences of polluted water as a result of rapid growth in their population, scarcity of water resources, and insufficient water treatment systems. Industrial wastewater contains a heavy load of metal ions and salts in addition to other organic pollutants. The carcinogenic and mutagenic nature of potentially toxic metals such as Pb, Hg, As, and Cd has already been classified as a priority pollutant [3,4]. These metals are distinguished toxicants even at negligible concentrations, which affect organelles involved in detoxification, the DNA repairing system, and metabolism [5,6]. Rapid growth in industrialization and unchecked discharge systems are adding toxic metals into water bodies and damaging the aquatic environment [7,8,9]. 

The World Health Organization (WHO) considers mercury (Hg), arsenic (As), lead (Pb), and cadmium (Cd) as the top listed potentially toxic metals associated with public health concern [10]. The main sources of Pb contamination in water, soil, and air are electrochemical industries, petrochemical industries, textile dyeing units (Pb salts as mordents), household water supply systems, and automobile exhausts [4,5]. Industrial effluent containing mercury, arsenic, and lead can inflict irreversible damage to the environment and human health [6,7,8]. This burning issue has led to the inspiration for a rising number of studies on the treatment of wastewater for the removal of potentially toxic metals like Pb, As, Hg, and Cd [9,10]. 

Among the potentially toxic metal series, inorganic lead (Pb) is considered neurotoxic because of its injurious effect on vital organs, enzyme systems, and the neurotransmission chain in humans [11]. Lead ^82^Pb= [Xe] 4f^14^ 5d^10^ 6s^2^ 6p^2^ is the 38th most abundant element in the earth’s crust, and its ionic forms, such as Pb (II) with halides, monoxide, and Pb (IV) chalcogenide and dioxides, are stable compounds in the environment [12]. Lead mining, smelting, battery manufacturing, water pipes, and the recycling and discharging of waste are common in several parts of the world. Therefore, lead (Pb-II) exposure is a wide-reaching and challenging issue in the world [13]. Even in natural water systems, the toxicity of Pb (II) has been detected all over the world, and WHO has established 0.05 mg/L of Pb (II) as the standard permissible limit for drinking water [14]. The Environmental Protection Agency (EPA) has specified an even lower level of Pb (II) in drinking water (0.01 mg/L) [15]. Therefore, the removal of Pb (II) from water is very much crucial for the protection of both human health and the environment [16,17,18]. Among diverse physical separation techniques, ion-exchange sorbents are considered the most viable materials due to selective and least-expensive characteristics for wastewater treatment [19]. Natural and synthetic zeolites had shown efficient chemothermal stability and potential application as toxic gas absorbers [20] and selective ion adsorbents [21]. 

The synthesis of zeolite from chemical sources like pure aluminates and silicates is expensive. Zeolite manufacturers are in search of economical raw materials for zeolite synthesis to minimize the production cost [21,22]. On the other hand, the disposal of fly ash is an economic and ecological problem for every country [22]. The hydrothermal process is common for the synthesis of zeolites using fly ash, but the alkaline fusion process, in which fly ash and NaOH are fused at high temperature to form Na-zeolites, can reduce the reaction time, with improved structural characteristics of the final product [23]. A multistep procedure can offer more advantages for the extraction of Si from fly ash using an alkaline treatment, followed by a synthesis of active zeolites by adding an Al-based precursor [24]. The modification of cheap raw materials into unique mesoporous Na-zeolites could be a feasible approach for the effective removal of toxic Pb (II) ions from wastewater [25]. Rag fly ash can be obtained as waste from ragged clothes-based boiler plants of textile industries. This research work is designed to use rag fly ash as a new class of raw material for the synthesis of value-added adsorbents like Na-zeolites for sequestration of toxic metal (Pb-II) from synthetic wastewater.

## 2. Materials and Methods

Rag fly ash (RFA) was collected using a striated sampling technique from the boiler unit of Ahmad Sizing Industry, Faisalabad, Pakistan. Hydrochloric acid (HCl 37% Sigma Aldrich, USA), sodium hydroxide (NaOH, 99%, Merck, USA), sodium aluminate (NaAlO_2_, 98%, Sigma Aldrich, USA), sodium silicate (Na_2_SiO_3_.9H_2_O, 99%, Merck, Germany), and PbCl_2_ (98% Merck, Germany) were of analytical grade and used as received.

### 2.1. Pretreatment and Physicochemical Properties of RFA

Fly ash samples were powdered and sieved under dry conditions using an 80-μm mesh size sieve to eliminate coarser particles, leaving fine ash particles ranging from <150–200 μm in diameter [26]. The chemical composition of the fly ash sample for major oxides was determined using an X-ray fluorescence spectrophotometer (XRF-PW 148, Philips, Netherland), following the standard method of characterization [27]. The leachability of the potentially toxic metals from RFA was evaluated by soaking the RFA in deionized water (DiW) for 24 h, and their concentrations were analyzed by ICP-OES (Prodigy 7, Teledyne Leeman Labs, Netherland). The RFA sample (0.5 g) was dissolved in 100 mL of DiW and stirred well for 24 h at 30 °C and then subjected to a leachability evaluation and pH and electrical conductivity (EC) measurements by multimeter (Multimeter HI-9811-5, Henna, Italy) [28]. Similarly, specific gravity (SG), loss of ignition (LOI), and cation exchange capacity (CEC) values of RFA were assessed by following the standard procedures [29].

### 2.2. NaOH-Activated Synthesis of Na-Zeolites

**Figure 1:**
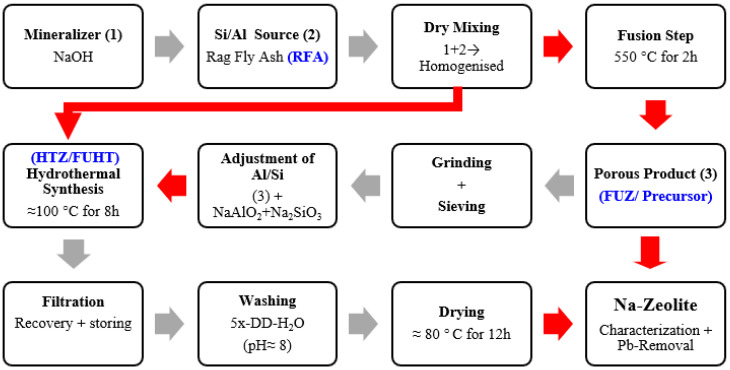
Flowsheet diagram for NaOH-activated hydrothermal (HTZ), fusion (FUZ), and fusion-assisted hydrothermal (FUHT) synthesis of Na-zeolites from rag fly ash (RFA).

In the conventional hydrothermal synthesis of zeolite (HTZ), 50 g of RFA was added to the NaOH (2 M) solution, keeping the 0.1 S/L ratio under dynamic conditions in a reactor equipped with a reflux condenser and an oil bath. The oil bath temperature was adjusted to 120 °C, resulting in a solution temperature of about 100 °C under atmospheric pressure, and cured for 8 h to get the HTZ product [30]. In the fusion synthesis of zeolite (FUZ), an alkaline fusion step was involved prior to the hydrothermal step, where 25 g of RFA was dry-mixed with 12.5 g of NaOH powder (2:1) for 30 min in a blend mixer. The resultant mixture was fused at 550 °C in oxidized conditions for two hours by adjusting the ramp rate in a muffle furnace (Muffle Furnace B-170, Nabertherm, Germany) [26]. In fusion-assisted hydrothermal synthesis (FUHT), 12.5 g of the fused product (FUZ) was dissolved in 125 mL of double-distilled water (DD-H_2_O) to form the amorphous precursor gel in a double-neck reactor under reflux conditions for hydrothermal treatment. For the adjustment of Si/Al, NaAlO_2_ and Na_2_SiO_3_.9H_2_O solutions were poured into the reaction vessel dropwise, keeping the solid-to-liquid ratio (1:10) constant. In FUHT synthesis, reaction conditions remained identical to HTZ synthesis [27]. After washing and neutralizing the residues up to pH = 8, the gel was dried at ≈80 °C for 12 h in a drying oven (Drying Oven TK/LG 154, Ehret, USA) [31]. The complete processes of Na-zeolite synthesis from RFA, employing all the applied techniques, are elaborated in a flow sheet diagram in Figure 1.

### 2.3. Characterization of RFA and Na-Zeolites

The sorption performance of RFA and Na-zeolites was determined by CEC measurement in SI units (meq/100 g of sample), following the ammonium acetate method [29]. The crystallinity and mineralogical compositions of RFA and Na-zeolites were studied by powdered XRD analysis (XRD System D-8-Advanced, Bruker, Germany) by subjecting them to Cu-Kα radiation. XRD data collection was conceded via 2θ in the range of 10–50°, with a scanning step of 0.02° [32]. Surface chemistry and functionality of synthesized products were studied by FTIR spectroscopy (FTIR Spectrophotometer, Spectrum-2, Perkin Elmer, Germany) by scanning the material sample in the range of 3500–450 cm^−1^ (the region of major spectral variation) in transmission mode [33,34]. Morphology of the RFA and Na-zeolite materials was probed by SEM (JSM-5910 Scanning Electron Microscope, JEOL, Japan) under the following settings: EHT = 15.00 kV, Signal A = SE1, WD = 8.0 mm [35].

### 2.4. Sorption of Toxic Metal (Pb-II) from Wastewater

The sorption of Pb (II) by RFA and Na-zeolites was studied to examine their efficiency in cleaning synthetic wastewater. Pb (II) stock solution (10 g/L) was prepared by dissolving PbCl_2_ in deionized water (DiW) and further diluted for batch reactions [36]. Sorption tests were carried in 250 mL Erlenmeyer flasks, with constant stirring at 200 rpm at 25 °C. In these experiments, the initial metal ion concentration (100–300 mg/L), mass of Na-zeolites (0.25–1.5 g), pH (2–12), and time (10–90 min) of the heterogeneous mixture were evaluated by batch experiments. After the programmed time, each reaction vessel was removed from the shaker, and the supernatant collected by filtering the suspension was set apart in a refrigerator at ≈4 °C for ICP-OES analysis. The quantity of Pb (II) adsorbed by the different adsorbents was calculated using Equations (1) and (2) [14].

Qe = V (Co − Ce)/m(1)

R_em_ = (Co − Ce)/Co × 100 (2)

where “Qe” is the Pb (II) concentration (mg/g) adsorbed by RFA, HTZ, FUZ, and FUHT at equilibrium, “Co” and “Ce” are concentrations (mg/L) of Pb (II) in solution before and after treatment, respectively, whereas “m” is the mass (g) of adsorbents used and “V” is the volume (L) of heterogeneous solution [37]. Correspondingly, “R_em_” is the removal efficiency of the adsorbent for selected ions at equilibrium. The intact series of trials were carried in three replicates to evaluate test reproducibility under the same set of conditions [38].

## 3. Results and Discussions

### 3.1. Physicochemical Characteristics of RFA and Na-Zeolites

Extract of the RFA sample revealed little basic pH, while the supernatants of the fused products showed higher pH due to alkaline salt formation. Likewise, hydrothermal crystallization produced basic supernatant due to the addition of alkali and aluminosilicates in order to adjust the Al/Si ratio. Electrical conductivity (EC) measurement is a fast, inexpensive, and consistent way of measuring the ionic contents in a solution [39]. The EC of the deionized water was extremely low, while the EC of the RFA extract did not increase too much due to the existence of insoluble constituents in water. However, activation via NaOH in the hydrothermal and fusion steps produced alkaline salts with high mobility of the respective ions, which increased the conductance of the supernatants. Similarly, during FUHT, a number of electrolytes were augmented, which amplified the EC (215 μS/cm) at high temperature, whereas the solubility also affected thermodynamically. Loss on ignition (LOI) of RFA relates to the presence of carbonates, water in residual minerals, and the combustion of complimentary carbon [37]. In the present study, the loss of ignition of RFA was observed at 2.2%, which emphasized the F-type ash of low Ca contents, as per standards [37,39]. Carbon is the most important component of LOI, which is encapsulated in glassy crystallites during the incineration process. Fusion-based zeolite (FUZ) showed minimum LOI because the resultant product was fused at high temperature, and all the combustibles were lost. An increase in LOI of HTZ/FUHT, even after oven drying at 80 °C, is linked to the moisture content due to hydrothermal treatment. 

Adsorbents are recognized by their chief property, i.e., CEC, which determines their suitability for the removal of toxic metals from wastewater [27,40]. During the synthesis of Na-zeolites, the trivalent Al creates a deficiency of positive charge by replacing the tetravalent Si atoms that are responsible for its CEC [40]. Na^+^ balances the deficiency of charge from NaOH, considering that it is openly linked to the presence of Na-zeolites [27]. In the present study, the CEC (435 meq/100 g) of the Na-zeolites synthesized by fusion-assisted hydrothermal treatment was found to be superior, with better crystallinity. In fact, Na-zeolites derived from RFA were mesoporous materials with high CEC that could help facilitate the entrapment of toxic metal ions into mesoporous structure. It is evident from the results of this study (Table 1) that the CEC of RFA (78 meq/100 g) was very low, while alkaline modification enhanced it exponentially, which could lead to the sequestration of potentially toxic metals from wastewater. Statistical analysis showed significant (*p* < 0.05) differences in the CEC, LOI, and EC among the samples. 

RFA is generally composed of Al and Si, which can be aluminosilicate precursors in the synthesis of active zeolites [41]. It is evident that zeolite crystals vary in synthesized materials from fly ash material, compared to pure reagents. Zeolites (Na-zeolites) synthesized from fly ash have less reactivity of Si and Al due to the presence of other cationic impurities such as Fe, Ca, which may cause a hindrance in crystallization [42]. The presence of iron oxide (Fe_2_O_3_) may decrease the attractiveness of the product by incorporating metallic ions into the zeolite matrix, forming a brownish tinge [43]. Generally, fly ash containing less than 20% of CaO constituents is categorized as class “F” type fly ash [44]. The present study shows that RFA is a unique and new class of “F” type ash, comprising Al_2_O_3_, SiO_2_, F_2_O_3_, and CaO in the form of quartz, mullite, and hematite, along with calcite, inorganic lime, and sulfate [45]. It is wise to analyze the chemical composition and leaching features of raw fly ash prior to deposit or utilization [46]. In our study, RFA showed very low leaching of potentially toxic metals as an indicator of little hazardous effect on soil and underground water [47,48,49]. The chemical analysis of RFA showed significant (*p* < 0.05) differences in metallic oxide constituents. RFA used for the synthesis of Na-zeolites was mainly composed of SiO_2_ (60%) and Al_2_O_3_ (23%), along with other minor constituents of TiO_2_, Fe_2_O_3_, and CaO. Mineral profiles of supernatants of RFA revealed the leachability of metals, which are presented in Table 2 with their concentrations (ppm). It was observed that the leaching trend of various metals from RFA was significantly (*p* < 0.05) different. Maximum leaching was observed for Al, Mg, Na, and K, whereas Pb and Ti were found to have the lowest leaching. Overall, the compositional attributes of RFA, especially in terms of metal oxides, show its suitability to be recycled and, most probably, for the synthesis of value-added products like zeolites/adsorbents for multifaced applications.

### 3.2. Development Process and Crystal Growth Mechanism of Na-Zeolites

Generally, low calcium “F” type fly ash contains quartz (≥99% SiO_2_), glass, and mullite (≥27% SiO_2_ ≥70% Al_2_O_3_) as the major crystalline compounds. In contrast, high calcium fly ash comprises high amounts of mullite, calcium silicate, tricalcium silicate, and tetra calcium aluminosilicate, which are reluctant to dissolve in the solution even at high pH and hinder the crystal growth process during zeolites formation [50]. Active aluminates and silicates from fly ash are dissolved readily during alkaline hydrothermal reactions and participate effectively in zeolite derivation. The constituents of fly ash dissolved at 20–120 °C in the initial step, in the presence of OH^−^ in basic solution, have imperative involvement in the zeolite crystallization process [51]. The glassy phase of fly ash can play a leading role during zeolite synthesis because it is dissolved easily into the alkaline solution in the fusion process. The fusion process is considered superior, owing to the advantage of dissolving the hematite, quartz, and mullite phases of fly ash, whereas these persist in the final product in the hydrothermal process [52]. The addition of alkali to the raw material acts as an activating/mineralizing agent during the fusion process. Zeolite yield from the fusion process can be as high as (±90%) because a number of crystalline phases in the fly ash can react during the calcination step. Fusion also increases the surface area of the product 10 times compared to fusion-free crystallization [53]. High-temperature synergic fusion and hydrothermal activation of fly ash generate active forms of Al and Si in the synthesis process of Na-zeolites [27,54]. An alkaline fusion step, followed by a hydrothermal process, facilitates the formation of active Na-zeolite material that is easily soluble in water and speeds up the zeolite crystallization process [54]. The fusion step also contributes to improving the porosity of the synthesized materials, which can increase the removal efficiency of the modified Na-zeolites [27].

The chemistry of interconversion of fly ash into zeolite is quite complex due to a number of factors involved. The mineral structure of fly ash is destroyed during fusion and hydrothermal curing, which leads to the easy dissolution of mullite and quartz minerals in the reaction mixture [55]. In general, dissolution depends significantly on the pH of the medium in the presence of the mineralizing agent (NaOH). As the hydroxyl ion (OH^−^) concentration increases, the crystalline phases of fly ash dissolve easily, forming a salt of sodium aluminosilicate in the alkaline fusion process. In the next phase of zeolitization, the dissolved Na-aluminosilicate salts of fly ash release ionic species that are further transported to the nucleation sites. Thus, the dissolution of insoluble minerals in fly ash residue introduces aluminosilicate into the reaction gel and ultimately increases the conversion rate of fly ash into Na-zeolite. During the nucleation step, the gel composition is considerably affected by kinetic and thermodynamic parameters [56]. During nucleation, different nucleation sites are established, following the Ostwald rule. According to this rule, larger and stable crystallites destabilize the smaller and minor coexisting crystals of the competing phase, decreasing crystal size distribution and encouraging the growth of the metastable phases of zeolite [56,57]. With prolonged reaction time, stable-phase crystals form at the expense of the primary metastable phases. Eventually, the stable phases reach the necessary equilibrium conditions to stimulate the crystal growth of the final zeolite materials [57]. Ultimately, zeolitization forms the zeolite originators, which contain tetrahedrons of “Si” and “Al” arranged haphazardly. These tetrahedrons are evenly distributed along the polymeric chains that are cross-linked to arrange cavities adequately to accommodate the charge-balancing Na ions. To sum up, crystallization involves the assimilation of particles from the solution by a growth process, and Na-zeolite crystals start to grow and reach a critical size as porous materials [30,58]. The condensation of aluminates and silicates is also accomplished by forming an aluminosilicate gel on the surface of fly ash residues by precipitation [59]. Chemically, the inactive ᾳ-quartz and inert mullite crystallites react with NaOH, according to the following empirical equations, to produce active forms of silicates (mainly) and aluminates (scarcely).

SiO_2(s)_ + 2NaOH_(s)_ → Na_2_SiO_3(s)_ + H_2_O (3)

Al_2_O_3(s)_ + 2NaOH_(s)_ → 2NaAlO_2(s)_ +H_2_O (4)

nNa_2_SiO_3(s)_ + nNaAlO_2(s)_ + _y_H_2_O → Na_2_O.Al_2_O_3_._x_SiO_2_._y_H_2_O (5)

Here, “n” is a whole or fractional value, while “x” varies from two to ten but is mostly ≥ 2; “y” is the molecules of water voids in the Na-zeolite, which fluctuates from two to seven [48,60]. Al_2_O_3_ and SiO_2_ form the cationic framework since they form the tetrahedral crystalline framework with oxygen atoms [49,61]. By and large, it is acknowledged that no double AlO_4_^¯^ are attached by mutual sharing their corner in the framework of zeolite, and it is established, based on the Lowenstein’s rule, that Al-O-Al linkages are not allowed and y/x ≥ 1 [62]. In absent or incomplete fusion treatment, silicate impurities remain in the final zeolite product even after drastic curing [63]. The final product after alkaline hydrothermal treatment may vary between Zeolite-A (Na-A) [64], Zeolite-X (Na-X) [55], Na-P1 [65], and hydroxy sodalite (SOD) [30], depending on temperature, pressure, time, Si/Al ratio provided, pH, and seeding of the reaction mixture.

### 3.3. Surface Chemistry and Functionality of RFA and Na-Zeolites

**Figure 2:**
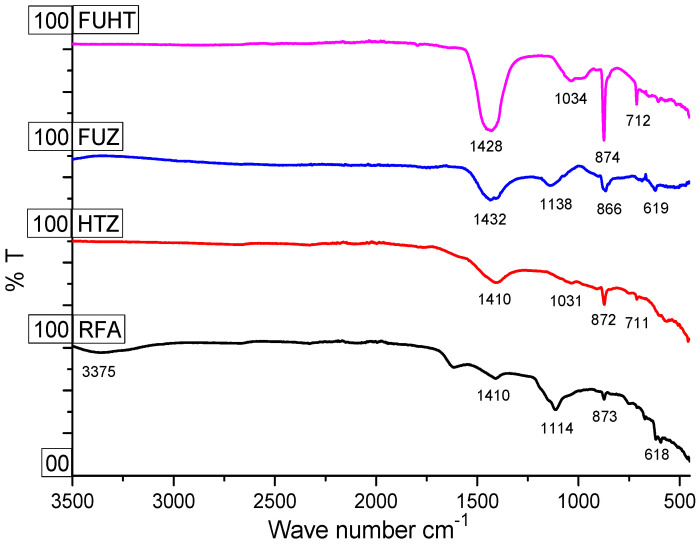
FTIR (ATR) spectra of RFA, hydrothermal synthesis of zeolite (HTZ), fusion synthesis of zeolite (FUZ), and fusion-assisted hydrothermal synthesis (FUHT), showing variation in functionality and surface chemistry.

The FTIR spectra of the RFA and Na-zeolites obtained after hydrothermal (HTZ), alkaline fusion (FUZ), and fusion-assisted hydrothermal (FUHT) treatments are given in Figure 2. The transmittance spectra showed remarkable differences between Na-zeolite products and RFA. Typical zeolite bands were observed in the FTIR spectra in contrast to RFA. The bands in the region of 450–500 cm^−1^ are attributed to inner tetrahedron vibrations of Si-O and Al-O or (T-O bands), also termed the bending vibrations of the Si/Al/-O bond of zeolite materials. The double ring vibrational bands occurring at 560–670 cm^−1^ indicate the (Al-O-Al) and (Si-O-Si) symmetric stretching of aluminates and silicates [26,27]. Sharp and small dips at 619 cm^−1^ of FUZ demonstrate (Al-O-Al) and (Si-O-Si) symmetric stretching vibrations in synthesized zeolite from RFA by the fusion process. Internal symmetric stretching vibration bands in the range of 720–610 cm^−1^ were only observed in the FUHT sample at 712 cm^−1^, which can be attributed to the internal vibrations of zeolites and can be assigned to RFA activation in the alkaline medium for the alkaline-activated product [26].

According to studies, bands appearing at 1100–1200 and 800–900 cm^−1^ can resemble quartz and mullite, respectively, which was observed in RFA (873 cm^−1^). The dips at 872 cm^−1^ in HTZ and the sharp but small peaks at 1138 and 866 cm^−1^ in FUZ show the constituents of unreacted quartz and mullite, while only an extra-large peak at 874 cm^−1^ in FUHT confirmed an incomplete but progressive conversion of RFA minerals into Na-zeolites. The peaks at 950–1000cm^−1^ are assigned to external symmetric and internal asymmetric stretching of T-O-T (T = Si, Al) and vitreous phases of RFA. The presence of bands at 1034 cm^−1^ in FUHT and a very small dip at 1031 cm^−1^ in HTZ shows T-O-T asymmetric stretching and confirms the presence of Na-zeolite phases in the final products.

The wide band appearing between 1350–1450 cm^−1^ is owed to the vibration of [SiO_4_]^4−^ and [AlO_4_]^5−^ components [66]. Similarly, a very short dip at 1410 cm^−1^ in RFA and extra-large and sharp peaks of HTZ (1410 cm^−1^), FUHT (1428 cm^−1^), and FUZ (1432 cm^−1^) indicate the presence of aluminates and silicates in the final Na-zeolite products. In FTIR spectra of the zeolites from RFA, vibration bands at 3200–3400 and 1600–1650 cm^−1^ are due to (H-OH) bending vibrations [67]. The bands at 3200–3400 and 1600–650 cm^−1^ indicate the presence of water, indicated at 3375 cm^−1^ in RFA, whereas the nonappearance of such dips in HTZ, FUZ, and FUHT show the absence of water in the synthesized Na-zeolites. The swing in absorption frequency is related to the compositional property that is directly linked to the Al/Si ratio and the conversion process of RFA into value-added Na-zeolites. Very clear functionality differences in the forms of bands and dips at important IR regions of synthesized samples and raw material spectra are evidence of the successful modification of zeolites. Overall, the interpretation of FTIR spectra of all three samples, in contrast to the RFA sample, provides clear evidence of the formation of our desired product, i.e., Na-zeolites, in which the maximum RFA-to-zeolite conversion rate was achieved using FUHT, followed by FUZ and HTZ.

### 3.4. SEM Micrographs

**Figure 3:**
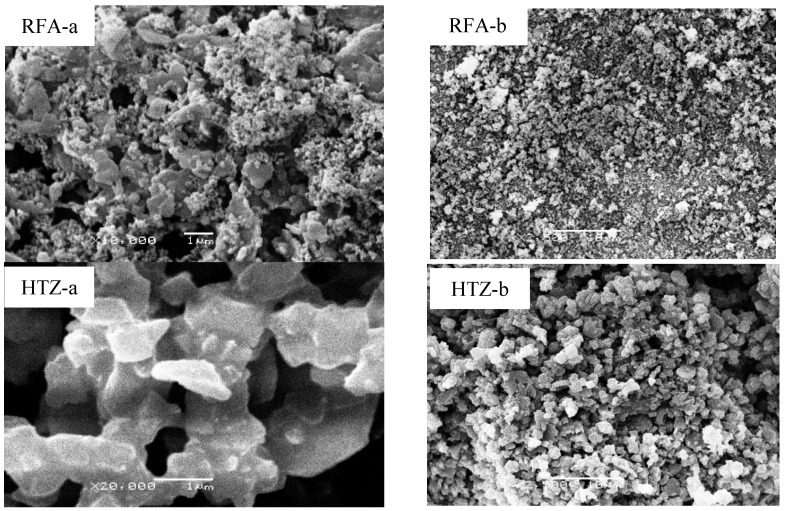

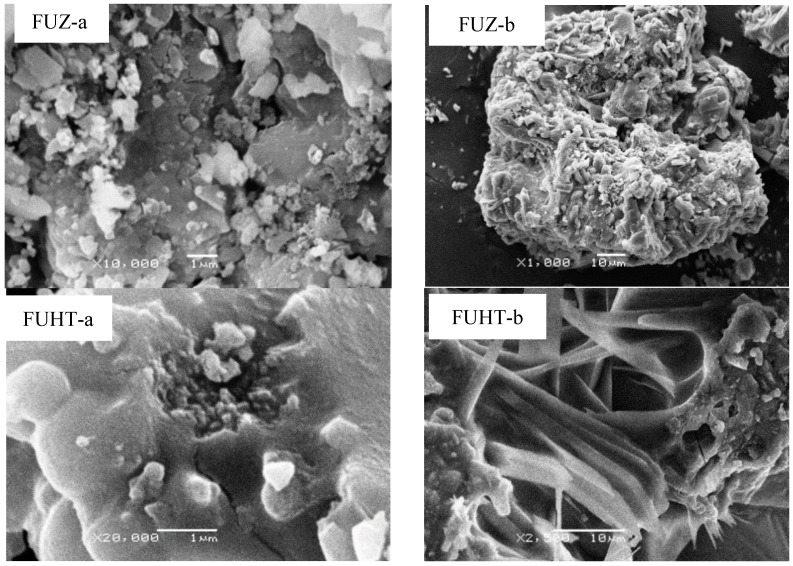
SEM micrographs showing variable morphologies of RFA-, HTZ-, FUZ-, and FUHT-based Na-zeolites derived from RFA under different magnification scales (a = 1 µm and b = 10 µm).

The morphology of raw material (RFA) and synthesized products is presented by SEM micrographs in Figure 3. The SEM micrographs show the irregular morphology of RFA in contrast to synthesized HTZ, FUZ, and FUHT samples. The micrographs also show the amorphous alumina and silica residues, describing the morphological conformation, even though quartz (A) and mullite (Q) phases occur in the unreacted glassy phase. It was observed that in conventional and synergic modification, the surface of Na-zeolites showed evident changes as alkaline hydrothermal curing produces NaP1 and SOD [27,65]. In the fusion process, high temperature affects the morphology of Na-zeolites as Na-X, whereas fusion-assisted hydrothermal synthesis decreases the formation of Na-X and converts it into NaP1, a SOD-type zeolite [30,41]. Nonuniform size distribution of phases was obtained by fusion under static and oxidized conditions. Variation in the morphologies of the products indicates that as the temperature rises in the fusion process, zeolitization proceeds abruptly, and a narrow range of shapes and sizes of zeolite material develops. In this study, fine grains with homogeneous, spherical, and porous morphologies were observed in HTZ and FUZ products, having grain sizes less than 0.5 µm (Figure 3). Sharp needle-like phases of FUHT were obtained by treating the fused products hydrothermally. The spherical cenospheres generally show a large variation in their dimensions and are supposed to form by the release of H_2_O vapors and CO_2_ gas, evolved from minerals within the burning materials [68,69]. Fly ash after treatment and purification forms specific shapes, such as rectangular and cubic geometries for Na-P1 and Na-A, while agglomerates resemble SOD and Na-X types of Na-zeolites [49,69,70,71,72]. 

### 3.5. XRD Analysis

**Figure 4:**
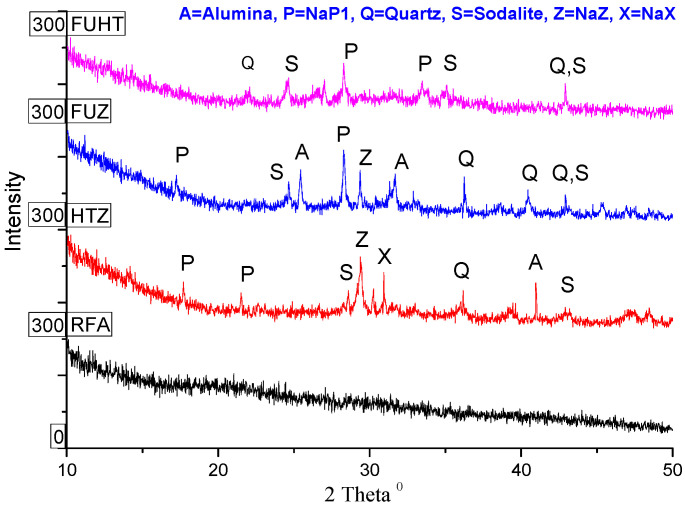
XRD crystallograms of RFA and its derived Na-Zeolites.

The XRD patterns in Figure 4 reveal that RFA is largely composed of minerals of Al and Si in different proportions of alumina and silica, which is also reinforced by evidence from XRF analysis. The XRD outcomes back up the fact of HTZ, FUZ, and FUHT synthesis of Na-zeolites as the derivatives of RFA. Some uncommonly weak peaks signify the existence of quartz (SiO_2_) and mullite (Al_6_Si_2_O_13_) as crystalline phases in RFA, which can be attributed to the melting at high temperature during the course of rag clothes combustion. The peaks of crystalline phases of alumina (A), quartz (Q), NaX (X), NaZ (Z), NaP1 (P), and hydroxyl sodalite (S) are present in the synthesized products. It is evident that crystalline phases of Na-zeolites are developed by alkaline fusion with NaOH, as compared to RFA, where no peaks of aluminates and silicates were observed. Fusion-assisted hydrothermal treatment was found to be superior compared to the single-mode fusion technique, which showed NaX (X) and SOD (S) along with unreacted glassy phases like quartz (Q) and alumina (A). High yields of good quality zeolite product could be achieved by sensible choices of pH, temperature, reaction time, and synthesis technique [73]. It is observed that the highest Na-zeolite yield could be obtained from high concentrations of amorphous SiO_2_, and percentage yield decreases due to the presence of nonreactive hematite and magnetite phases and stabilized forms of silica such as mullite and quartz in raw materials [74]. These silica and alumina minerals in raw materials lead to the dominant conversion of sodium silicate (Na_2_SiO_3_) and sodium aluminosilicate (NaAlSiO_4_) as Na-zeolites [27]. Conversely, in some cases, a high degree of well-size crystalline phases of fly-ash-based zeolites could be obtained with alkaline activation, using NaOH as the activator [43]. A comparison between different synthesis techniques suggests the feat of various mechanisms directing the synthesis of the diverse inorganic phases in fly-ash-based zeolites [75].

### 3.6. Applications of Fly-Ash-Based Na-Zeolites for the Removal of Potentially Toxic Metals

Toxic metals generate environmental anxiety due to their tendency for bioaccumulation and their nonbiodegradable nature and carcinogenicity [37]. The Union Carbide Corporation synthesized zeolite in a hydrothermal process and arranged a chain of zeolites, consisting of Na-zeolites like Na-X, Na-Y, and Na-A, as new porous sorbent materials from fly ash for the decontamination of wastewater from a number of potentially toxic metals. Removal of some potentially hazardous metal series by fly-ash-based zeolites in previous work [37,50,76,77] is documented in Table 3.

Raw fly ash gets rid of <8% of lead (Pb^2+^); however, its modified counterpart Na-zeolite showed up to 98% removal efficiency [78]. In a number of studies, toxic metals such as Pb^2+^ [79], As^3+^ [80], Cr^3+^ [52], and Cu^2+^ [81] were successfully removed from wastewater using fly-ash-based zeolites as sorbents. The adsorption process has always been the preferred method for the removal of toxic metal from water [82]. Fly-ash-based Na-zeolites provide new active sites on their surface for metal complex formation in a basic environment [83]. The presence of functional groups like (SiO_4_^−^) and (AlO_4_^−^) in zeolites could develop an electrostatic force of attraction with metallic ions, leading to the chemisorption process shown in Equations (6) and (7).

2(≡SiO^−^) + M^2+^ → (≡SiO)_2_M (6)

2(≡AlO^−^) + M^2+^ → (≡AlO)_2_M (7)

Factors such as pH, type and amount of adsorbent, nature and initial concentration of metal ion, time, and the presence of competing and complexing ions determine the removal efficiency of the adsorbent [84]. In the present investigation, experimental conditions viz pH, mass of adsorbent, initial metal ion concentration, and time were studied, and a detailed discussion is given below.

#### 3.6.1. Effect of pH on Removal Efficiency of Pb (II) by Na-Zeolites

**Figure 5:**
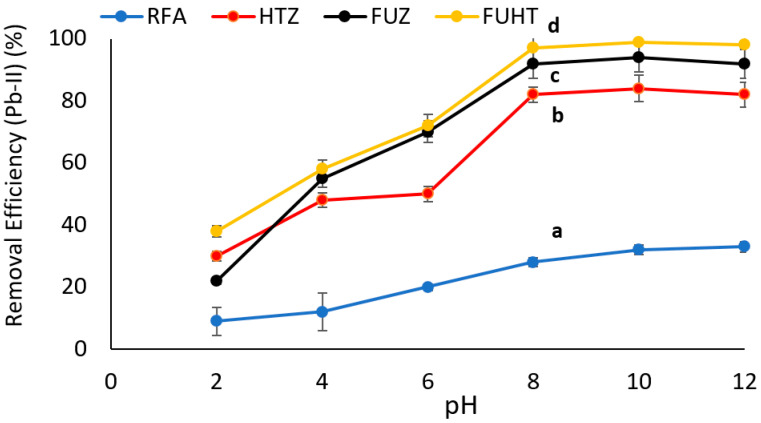
Effect of pH on the removal efficiency of Pb (II) at 100 mg/L metal ion concentration, 1 g/100 mL mass of adsorbent (RFA and Na-zeolites), and 30 min contact time at 25 °C. Notes: a, b, c, d shows significant (*p* < 0.05) differences among different zeolite samples.

The percent removal efficiency of Na-zeolite is directly linked to the pH of the medium for chemisorption of potentially toxic metals. In this work, the effect of pH (2–12) on (Pb-II) adsorption by RFA and Na-zeolites was investigated using 1 g mass of adsorbent with an initial metal ion concentration of 100 mg/L for 30 min of reaction time. Data presented in Figure 5 show that by increasing the pH of the heterogeneous solution, the metal removal efficiency increased, and maximum removal efficiency (~98%) was recorded at pH 8 by FUHT zeolites. After that, by increasing the pH from 8 to 12, there was no significant improvement in metal removal efficiency, showing the steady adsorption behavior of the derived Na-zeolites. Statistical analysis showed significant (*p* < 0.05) differences (mentioned by letters i.e., a, b, c and d) among Na-zeolites, as well as RFA, with a dominating removal of Pb (II) by FUHT, i.e., 98%, and the lowest removal of RFA. Our findings are in close agreement with those reported in previous studies, which also claimed 6–8 pH as the most suitable and optimum pH for metal ion sequestration [85,86,87]. Some previous studies have revealed that negatively charged compounds of Pb (II) are augmented, compared to positively charge compounds, by increasing the pH of the medium, and for the successful sequestration of metal ions, the pH of the solution should be near 6–8 [88]. On the other hand, a solution with pH ≤ 4 contains high concentrations of H^+^ ions that interfere with soluble metal cations and surface sites of adsorbents, and thus, the similar ions are in competition for attachment, which leads to a reduction in metal removal from wastewater [89]. However, with an increase in pH of the medium, adsorption of cations often increases because negatively charged surfaces of adsorbents attach firmly and instantly with toxic metal cations [90]. At higher pH (≥12), precipitation is the leading phenomenon due to the formation of insoluble hydroxides of respective metals compared to the adsorption process [91,92].

#### 3.6.2. Effect of Mass of Adsorbents on Removal Efficiency of Pb (II) by Na-Zeolites

**Figure 6:**
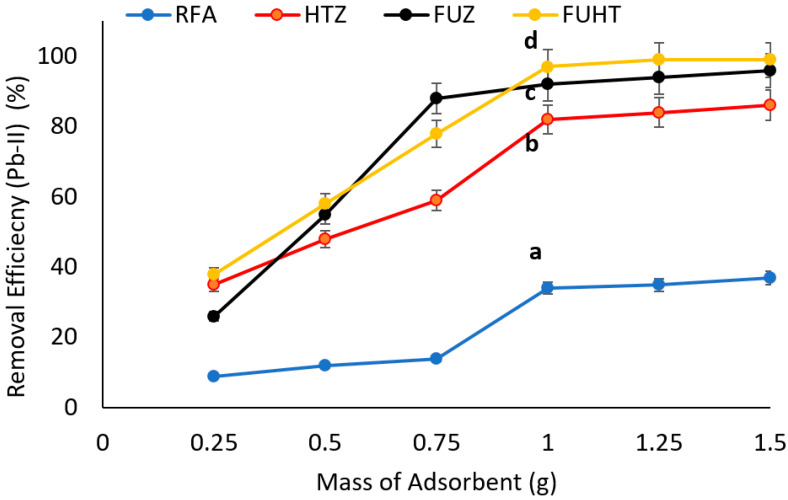
Effect of dose of adsorbents (RFA and Na-zeolites) on removal efficiency of Pb (II) at 100 mg/L metal ion concentration, pH ≈ 8, and 30 min contact time at 25 °C. Notes: a, b, c, d shows significant (*p* < 0.05) differences among different zeolite samples.

The effect of adsorbent dose (0.25 to 1.5 g of RFA and Na-zeolites mixed with synthetic polluted solution) on adsorption was investigated at pH 8 and an initial metal ion concentration 100 mg/L for 30 min; the data recorded is presented in Figure 6. The results revealed that with an increase in the dose of adsorbent, the removal of Pb (II) also increased and reached a maximum of 98% at 1 g of adsorbent (FUHT); there was no further improvement by increasing the dose of adsorbent. Statistical analysis showed significant (*p* < 0.05) differences (mentioned by letters i.e., a, b, c and d) between Na-zeolites as well as RFA, with a dominating removal of Pb (II) by FUHT, i.e., 98%, and the lowest removal of RFA. The findings of the present study are in agreement with literature reports that show the equilibrium concentration in the solution phase declines with increasing adsorbent mass at a fixed initial lead (Pb-II) concentration; meanwhile, the removal efficiency from wastewater increases with a higher Na-zeolite dose. This development is expected as the adsorbent dose offers higher surface area and more active sites of the Na-zeolite [93]. The percentage removal increases steadily with a further increase in the mass of adsorbents. It is submitted that Pb (II) ions adhere effectively due to the abundance of active sites on the surface of Na-zeolites, leading to a decrease in sorbate concentration in the solution [94]. For that reason, for our next trial, the ideal dose of adsorbent, i.e., 1 g/100 mL, was used.

#### 3.6.3. Effect of Metal Concentration on Removal Efficiency of Pb (II) by Na-Zeolites

**Figure 7:**
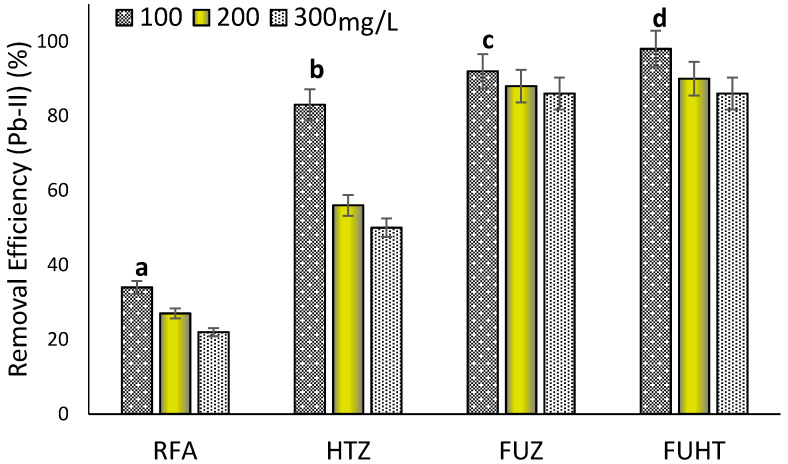
Effects of metal ion concentration of Pb (II) on its removal efficiency at pH ≈ 8, 1 g/100 mL mass of adsorbent (RFA and Na-zeolites), and 30 min contact time at 25 °C. Notes: a, b, c, d shows significant (*p* < 0.05) differences among different zeolite samples.

The effect of initial metal ion concentration was studied for RFA and Na-zeolites for the removal of Pb (II) from synthetic wastewater. The laboratory experiments were accompanied by fixed parameters—pH ≈ 8, 1 g/100 mL of adsorbents (Na-zeolites and RFA)—and fixed reaction time for 30 min by varying the initial Pb (II) concentrations, i.e., 100, 200, and 300 mg/L. Most of the Pb (II) ions present in the mixture interact with the active sites of the adsorbents, facilitating adsorption for 100 mg/L. It can be observed from the bar graph in Figure 7 that for 100 mg/L initial metal ion concentration and 30 min of reaction time, the removal efficiency for RFA = 34%, HTZ = 83%, FUZ = 92%, and, for FUHT, it was (amazingly) 96%. Hence, it shows that FUZ and FUHT are comparatively better adsorbents for the maximum removal of potentially toxic metal ion (Pb-II) in 30 min, as compared to RFA and HTZ. The synthesized Na-zeolites from RFA are proficient in reducing the Pb (II) concentration well below the threshold limit given by WHO (<0.05 mg/L). Statistical analysis showed significant (*p* < 0.05) differences (mentioned by letters i.e., a, b, c and d) between Na-zeolites as well as RFA for the removal of Pb (II) from synthetic wastewater in this study. It is because, at lower concentrations of cations, the number of metal ions present in the heterogeneous solutions interacts with the active sites of adsorbents assisting in prominent adsorption [95]. The systems with lower initial concentrations attain equilibrium in shorter periods, and the removal efficiency of the sorbent can be high [94]. The reason behind the process is the availability of more exchangeable sites at the Na-zeolite surface, with fewer numbers of sorbate ions [96]. On the other hand, at very high initial adsorbate ion (Pb-II) concentration, the active sites on the adsorbent surface become saturated, and therefore, these adsorbents (Na-zeolites) are unable to remove the metal ions effectively from wastewater [97].

#### 3.6.4. Effect of Time on Removal Efficiency of Pb (II) by Na-Zeolites

**Figure 8:**
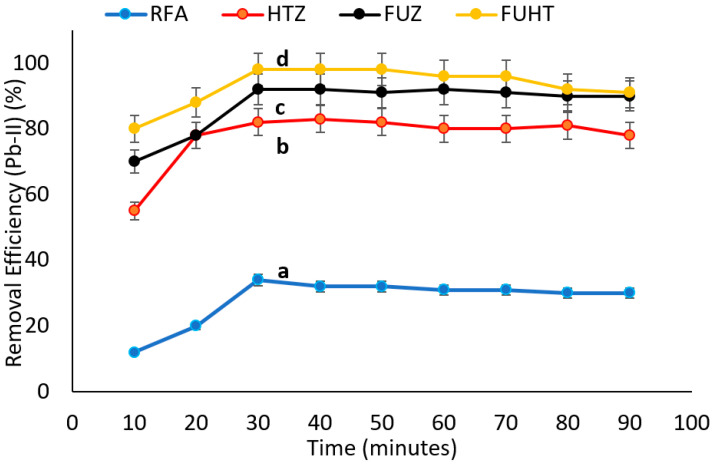
Effects of time variation on removal efficiency of Pb (II) at pH ≈ 8 and 1 g/100 mL of mass of adsorbent (RFA and Na-zeolites) at 25 °C. Notes: a, b, c, d shows significant (*p* < 0.05) differences among different zeolite samples.

The effect of time (10–90 min) was investigated on the removal efficiency of RFA and Na-zeolites at the previously selected parameters, i.e., dose of adsorbent 1 g/100 mL, pH 8, and 100 mg/L initial metal ion concentration. The data presented in the line graph (Figure 8) revealed that maximum Pb (II) removal efficiency (97%) was achieved in 30 min with the FUHT adsorbent and the minimum (35%) with RFA. Statistical analysis showed significant (*p* < 0.05) differences (mentioned by letters i.e., a, b, c and d) between Na-zeolites as well as RFA, with a dominating removal of Pb (II) by FUHT, i.e., 97%, and FUZ, i.e., 92%. At an increased time period for sorption with lower Pb (II) ion concentrations, active sites and mesopores of adsorbents are responsible for the adsorption and entrapment of metal ions at room temperature, i.e., 25 °C [98]. With the passage of time, the metal removal process reaches a saturation point, where the limited active sites on the adsorbent are available for the removal of Pb (II) ions. The mechanism studied by different researchers for metal ion chemisorption on adsorbent composites suggests surface complex modeling [99]. At the start of the sorption process, a high number of active sites are available at adsorbent surfaces, and a high amount of metal ions increases the rate of sorption. After 30 min of the sorption process, the active sites of the Na-zeolite surface decreases, and Pb (II) ions reach a lower number, which make them unable to improve the removal efficiency of the adsorbent. The contact time during the sorption of Pb (II) removal from wastewater shows rapid adsorption initially, and, thereafter, it decreases gradually or remains constant to achieve equilibrium in a shorter period of time, about 20–30 min. A further increase in contact time has an inverse and nonsignificant effect on removal efficiency [97]. As the mesopores of the synthesized Na-zeolite adsorbents get saturated, they restrict the further diffusion of Pb (II), which reduces the significance of prolonged contact times. The plots reveal that maximum percentage removal was highest during the first 30 min of adsorption, as a large surface area and active adsorbent sites were available. At the starting stage of adsorption, the rate of Pb (II) uptake is due to the transportation of charged species into the interior of the cage structure of fly-ash-based Na-zeolites [100].

### 3.7. Adsorption Isotherms

The Langmuir isotherm predominantly refers to monolayer adsorption phenomena over the homogenous surface of an adsorbent, while the Freundlich isothermal model explains the reversible and multilayer uptake of adsorbate for heterogeneous adsorption mechanisms [14]. The linear form of Langmuir and Freundlich isotherm models are given in Equations (8) and (9), respectively.

Ce/Qe = Ce/Qm + 1/Kl Qm(8)

Log Qe = log Kf + 1/n log Ce(9)

In Equation (8), “Ce” is the amount of Pb (II) (mg/L) at equilibrium, “Qe” is the uptake capacity (mg/g), while Qm is the possible monolayeric coverage of the adsorbent (mg/g) and Kl is the Langmuir constant (L/mg) that specifies the adsorption energy. The values of Kl and Qm were calculated from the slope and intercepts of the line of “Ce/Qe” versus “Ce”, respectively [101]. Similarly, in Equation (9), “Qe” is uptake capacity (mg/g), “Ce” is the amount of Pb-II at equilibrium (mg/L), while “Kf” and “n” are “Freundlich constants” regraded as adsorption capacity (mg/g) and adsorption intensity of the adsorbate on the adsorbents, respectively. The values of “n” and “Kf” can be calculated from the slope and intercept of the linear plot of “log Qe” versus “log Ce” [102].

Langmuir and Freundlich adsorption constants for the removal of Pb (II) from wastewater verified the significant adsorption of the adsorbate on Na-zeolites and RFA surfaces. Na-zeolites synthesized from RFA showed cost-effective activity as an adsorbent and its ability to reduce Pb (II) ion concentrations even below the permissible values (0.05 mg/L) established by WHO. Langmuir and Freundlich constants and regression (R^2^) values are mentioned in Table 4. The results from R^2^ values are best fitted by Langmuir (>96) and Freundlich (>98) isotherms; hence, it can be concluded that the adsorption of Pb (II) to Na-zeolites is a hybrid process rather than a monolayer adsorption mechanism. The Langmuir isotherm elucidates homogeneous monolayer adsorption, with no transmovement of the adsorbate in the plane of the adsorbent, while the Freundlich adsorption isotherm is nonideal and explains the mechanism for the multilayer deposit formation of toxic metal ions on adsorbents [103]. Removal of Pb (II) through adsorption via time versus removal efficiency is the rate-limiting step proposed by different researchers that involves chemisorption and metal removal belonging to physicochemical interactions between Pb (II) and zeolite adsorbents [104].

### 3.8. Kinetics Studies

The experimental data acquired in the study elucidates the kinetic study of the inclusive adsorption system. Reaction kinetics was studied for the sorption of Pb (II) by RFA and Na-zeolites. The pseudo first-order kinetic describes the physical interface of Pb (II) with the adsorbent surface. The pseudo first-order model ascertains that Pb (II) adsorption on Na-zeolites is directly proportional to the available active sites at the surface of the adsorbents. The nonlinear differential form of this model is given in Equation (10). Similarly, the chemisorption phenomena of Pb (II) by RFA and Na-zeolites can be explained by pseudo second-order rate kinetics. The nonlinear differential form of the pseudo second-order rate equation is given in Equation (11) [105], where “Qe” and “Qt” are the quantities of Pb (II) adsorbed at equilibrium and time “t”, K1 and K2 are the rate constants for sorption process, following pseudo first- and second-order rate constants, respectively [106].

dQt/dt = k1 (Qe − Qt)(10)

dQt/dt = k2 (Qe − Qt)^2^ (11)

The calculated values of rate constants for pseudo first-order (K_1_) and pseudo second-order (K_2_), Qe, and regression (R^2^) for the adsorption of Pb (II) by RFA and their modified zeolites are illustrated in Table 5. Calculated pseudo first-order values were close to experimental values, which exposes that the sorption of Pb (II) was physicochemical in nature for RFA. On the other hand, the value of correlation factor (R^2^) for pseudo second-order is high (R^2^ > 0.99) for FUHT and RFA (R^2^ ≈ 92), which submits that the pseudo second-order rate model is best-fitting to the observed data and that the process of adsorption follows the chemisorption mechanism [107]. 

## 4. Conclusions

RFA, a solid waste from textile industry, was successfully modified into mesoporous Na-zeolites (i.e., NaZ, NaX, SOD, NaP1) by conventional (HTZ), fusion (FUZ), and synergic fusion-assisted hydrothermal (FUHT) techniques. The synthesized Na-zeolites were quite suitable material for the removal of Pb (II) ions from wastewater because of their porous structure and active adsorbent sites. However, the FUHT-based zeolites were more valuable (mesoporously) and more efficient adsorbents for the removal of Pb(II) from wastewater. A maximum Pb(II) removal efficiency of 98% was achieved by FUHT-based zeolites at 100 mg/L initial metal ion concentration, 1 g/100 mL mass of adsorbent, 8 pH, and 30 min contact time conditions. The Freundlich adsorption isotherm suitably described the reaction between all the adsorbents, i.e., RFA, HTZ, FUZ, and FUHT, and Pb(II) ions in heterogeneous solutions, following the hybrid mechanism but not monolayer surface phenomena. We emphasize that further research is required to optimize the use of RFA-based zeolites for the removal of heavy metals from industrial wastewater. This study also implies that industrial solid waste (RFA) can be utilized for the treatment of industrial wastewater as a green approach. 

## Figures and Tables

**Table 1:** Physicochemical properties of supernatant/material of RFA and Na-zeolites.

Sample	Treatment	pH	EC (ᶙS/cm)	LOI (%)	CEC (meq/100 g)	SG (25 °C)
DI H_2_O	N/A	7.0 ± 0.2 ^b^	20 ± 1 ^a^	N/A	0	1.00 ± 0.12 ^a^
RFA	N/A	6.5 ± 0.2 ^a^	110 ± 5 ^b^	2.2 ± 0.2 ^c^	78 ± 4 ^a^	2.06 ± 0.13 ^b^
HTZ	Hydrothermal	12.5 ± 0.4 ^d^	148 ± 4 ^c^	1.2 ± 0.1 ^b^	150 ± 6 ^b^	2.71 ± 0.12 ^d^
FUZ	Alkaline Fusion	11.1 ± 0.5 ^c^	210 ± 8 ^d^	0.01 ± 0.0 ^a^	440 ± 18 ^c^	2.22 ± 0.14 ^c^
FUHT	Fusion + Hydrothermal	13.0 ± 0.4 ^d^	215 ± 6 ^d^	1.1 ± 0.1 ^b^	435 ± 19 ^c^	2.27 ± 0.11 ^c^

The values are mean ± SD of three independent experiments; different letters in superscript represent significant differences (*p* < 0.05) among samples.

**Table 2:** Chemical analysis of the RFA used for synthesis.

**Metallic Oxides**	**SiO_2_**	**Al_2_O_3_**	**TiO_2_**	**Fe_2_O_3_**	**Na_2_O**	**K_2_O**	**CaO**	**MgO**	**Others**
Composition (% wt)	60 ± 3 ^f^	23 ± 3 ^e^	1.8 ± 0.1 ^a^	4.2 ± 0.2	1.6 ± 0.1 ^b^	1.0 ± 0.1 ^a^	2.6 ± 0.1 ^c^	0.8 ± 0.1 ^a^	5.0 ± 0.3 ^d^
**Leached metals concentrations**									
Leached metals	Si	Al	Ti	Fe	Na	K	Ca	Mg	Pb
Concentration (ppm)	3.5 ± 0.2 ^c^	38 ± 1 ^g^	0.6 ± 0.1 ^b^	0.4 ± 0.1 ^b^	40 ± 2 ^f^	20 ± 1 ^e^	6.5 ± 0.3 ^d^	30 ± 2 ^f^	0.005 ± 0.0 ^a^

The values are mean ± SD of three independent experiments; different letters in superscript represent significant differences (*p* < 0.05) within the same row.

**Table 3:** Applications of fly-ash-based Na-zeolites and their metal-removal potential.

S. #	Fly-Ash-Based Na-Zeolites	Source of Fly Ash	Potentially Toxic Metals Removal Series
1	ANA, PHI	Brazil	Pb^2+^ > Cu^2+^ ≈ Zn^2+^ ≥ Mn^2+^
2	CAN	Canada	Pb^2+^ > Cu^2+^ > Ni^2+^
3	FAU	UK	Fe^2+^ > As^5+^ > Pb^2+^ > Zn^2+^ > Cu^2+^ > Ni^2+^ > Cr^2+^
4	Na-P1	Brazil	As^5+^ > Mn^2+^ > Fe^2+^ > Cu^2+^ > Ni^2+^ > Zn^2+^
5	Na-P1	Spain	Fe^2+^ ≥ Cu^2+^ ≥ Pd^2+^ ≥ Cd^2+^ > Zn^2+^ > Mn^2+^ > Sr^2+^
6	Na-P1	South Korea	Pb^2+^ > Cu^2+^ > Cd^2+^ > Zn^2+^
7	Na-P1	Netherland	Ba^2+^ > Cu^2+^ > Cd^2+^ ≈ Zn^2+^ > Co^2+^ > Ni^2+^
8	SOD	India	Pb^2+^ > Cd^2+^ > Zn^2+^
9	Na-X	Thailand	Pb > Cu > Cd
10	Na-X, FAU	Japan	Pb > Cu > Cd > Ni

**Table 4:** Isothermal study for adsorption of Pb^2+^ on RFA and Na-zeolites.

Model	Parameters	RFA	HTZ	FUZ	FUHT
Langmuir Isotherm	Kl (L/mg)	0.142	0.367	0.682	0.703
	Qm (mg/g)	34.3	107.6	179.7	173.8
	R^2^	0.916	0.962	0.963	0.968
Freundlich Isotherm	Kf (L/mg)	26.28	34.67	51.96	50.02
	n	2.264	2.757	3.278	4.862
	R^2^	0.948	0.982	0.983	0.991

**Table 5:** Kinetics study for adsorption of Pb (II) on RFA and Na-zeolites.

Adsorbent	Pseudo First-Order Model			Pseudo Second-Order Model		
	K1 (min^−1^)	Qe (mg/g)	R^2^	K2 (g/mg.min)	Qe (mg/g)	R^2^
RFA	1.21 × 10^−2^	17.2	0.916	8.17 × 10^−4^	34.3	0.922
HTZ	1.54 × 10^−2^	46.8	0.973	4.17 × 10^−3^	130	0.981
FUZ	8.62 × 10^−3^	54.6	0.981	4.54 × 10^−3^	180	0.991
FUHT	7.65 × 10^−3^	59.7	0.980	3.57 × 10^−3^	178	0.994

## Data Availability

Not applicable.
